# Nurse-perceived workload, staffing, and patient turnover: An analysis of longitudinal routine data

**DOI:** 10.1016/j.ijnsa.2026.100671

**Published:** 2026-09-05

**Authors:** Michael Ketzer, Sarah N. Musy, Christos T. Nakas, Michael Simon

**Affiliations:** a Institute of Nursing Science, Department of Public Health, Faculty of Medicine, University of Basel, Switzerland; b University Psychiatric Clinics Basel, University of Basel, Switzerland; c Laboratory of Biometry, University of Thessaly, Volos, Greece; d University Institute of Clinical Chemistry, Inselspital, Bern University Hospital, University of Bern, Bern, Switzerland

**Keywords:** Inpatients, Longitudinal studies, Nursing staff, Personnel staffing and scheduling, Routinely collected health data, Workload

## Abstract

**Background:**

Nursing workload is a critical factor in ensuring safe, high-quality patient care and maintaining nurse well-being. While high workload has been linked to adverse outcomes for both patients and staff, its assessment remains challenging. Common approaches rely on activity- or dependency-based measures or professional judgement, yet these often overlook important subjective and contextual factors. Nurse-perceived workload, particularly at the shift level, offers valuable insights but has received limited attention in research and is rarely used to guide staffing decisions.

**Objective:**

This retrospective longitudinal study explored nurse-perceived workload on shift level and its associations with nurse staffing, patient comorbidity score and turnover.

**Design:**

Retrospective observational study using longitudinal shift-level routine data.

**Setting(s):**

Five internal medicine units in a large Swiss acute care hospital.

**Participants:**

131 registered nurses (RN).

**Methods:**

We analysed five years (2015–2019) of routinely collected data from five internal medicine units of a Swiss tertiary hospital. Nurse-perceived workload was documented at the end of each shift using a single-item rating. Staffing system data (e.g., shift assignments, working time) were merged with patient discharge data (e.g., diagnoses, demographics, admission). Descriptive statistics, multilevel logistic regression, and a gradient boosting model were applied.

**Results:**

High workload was classified in 9,646 of 42,035 nurse-shift observations (22.9%). The number of incoming patients showed the strongest positive association with high workload perception (OR 1.30; 95% CI: 1.26–1.33). Higher RN staffing relative to expected staffing was associated with lower odds of high workload (OR 0.84; 95% CI: 0.78–0.91). Patient comorbidities also showed a positive association (OR 1.07; 95% CI: 1.06–1.08), as well as the number of outgoing patients (OR 1.02; 95% CI: 1.004–1.04). The gradient boosting model showed limited predictive performance.

**Conclusions:**

This study is among the first to investigate nurse-perceived workload using extensive longitudinal routine data. The findings suggest that single-item ratings are a practical means of capturing workload perception at scale. High workload perception was common and was positively associated with patient inflow and comorbidity, while higher staffing levels were associated with lower odds of high workload. These results underscore the potential of integrating subjective workload data into staffing policy and workforce planning to better support nurses and maintain care quality.

**Registration:**

not registered.

**Social media abstract:**

Nurse workload isn’t just personal. It’s part of the system: 23% of nurse-shift observations were classified as high workload; odds were higher with admissions, complexity, and lower with better staffing.

**What is already known**

• High nursing workload linked to burnout, care rationing, and poor patient outcomes.
• Workload is often measured with indicators that overlook nurses' experience.
• Subjective workload data remain underused in staffing research and planning.

**What this paper adds**

• Nurses rated workload as high in 23% of nurse-shift ratings, even in a well-staffed Swiss hospital.
• Admissions and patient complexity were associated positively, staffing negatively with higher workload.
• Subjective workload is a system-relevant metric that can inform staffing models.

## Background

Health systems face continuous pressure from rising care demands and limited staffing. Nurses, central to care delivery, are frequently exposed to high workload due to aging populations, high patient turnover, and persistent staff shortages. Such workload is linked to job dissatisfaction, burnout, turnover intentions (Dall’Ora et al., 2020; Holland et al., 2019; Michaeli et al., 2024; Shin et al., 2018), and negative patient outcomes, including missed care, adverse events, and increased mortality (Dall’Ora et al., 2022; Needleman et al., 2011; Ross et al., 2023).

Measuring nursing workload is essential but challenging, as definitions and tools vary (Twigg and Duffield, 2009). In this study, workload is defined as the sum of tasks during a shift, including direct, indirect, and non-patient-related activities (Alghamdi, 2016; Swiger et al., 2016). Existing approaches include patient-to-nurse ratios, activity- or dependency-based methods, professional judgement, and self-reported measures. Each has limitations: ratios overlook patient complexity and turnover (Griffiths et al., 2018; Musy et al., 2020); activity- and dependency-based tools often miss indirect or cognitive demands; professional judgement can be inconsistent; and self-reports, while capturing lived experience, may be influenced by individual factors (Hoonakker et al., 2011; Twigg and Duffield, 2009).

Perceived workload, reported by nurses during or after a shift, captures direct and indirect duties and reflects how staff experience workload (Hoonakker et al., 2011). Despite its promise, empirical validation in clinical practice remains limited. Overtime, a frequent occurrence when care demands exceed staffing (Gehri et al., 2023; Watanabe and Yamauchi, 2018), may serve as an external indicator of workload, offering a way to test criterion-related validity of self-reported workload measures.

Most prior studies on workload rely on cross-sectional surveys, limiting generalisability. Analysing routinely collected shift-level data offers new opportunities to better understand workload in clinical practice. In this study, we aimed to: (1) describe nurses’ workload perceptions across hospital units; (2) examine criterion-related validity evidence of a single-item workload measure using overtime; and (3) identify factors associated with high perceived workload.

## Methods

### Study design and setting

We conducted a retrospective study using routinely collected data from five medical inpatient units in a large Swiss tertiary hospital between January 1, 2015, and December 31, 2019.

### Participants

All inpatients hospitalized on one of the five units during the study period were included, except those with stays under 24 h.

Registered nurses (RNs) were eligible if they had permanent employment, ≥80% workload, and ≥3 months of unit tenure. These criteria minimized confounding from orientation periods, part-time work, or temporary contracts. Excluded were temporary staff, RNs in supervisory roles, licensed practical nurses, aides, students, and administrative staff. We focused on RNs, who hold primary responsibility for patient care.

### Data sources and variables

Data came from the hospital’s electronic nurse staffing system and medical discharge records. The staffing system provided working hours, shift types, unit assignments, and workload ratings; discharge records included admissions, diagnoses, and demographics. Datasets were linked with unique identifiers and timestamps, and all identifying information was removed. Nurses rated perceived workload per shift on a 5-point scale from very low to very high. The unit of observation was the nurse-shift, defined as one eligible RN working one shift. A workload rating could be recorded for each nurse-shift observation. When multiple RNs worked the same unit shift, their nurse-shift observations were retained separately and were not aggregated. Shift-level variables, including patient census, turnover, comorbidity, and staffing, were therefore identical for nurse-shift observations referring to the same unit shift. Ratings above the midpoint were coded as high workload (1); the midpoint and below as not high workload (0). Missing workload ratings were coded as not high workload in the primary analysis to retain all eligible nurse-shift observations. As this approach assumes that an unrecorded rating does not indicate high workload, we assessed its robustness in a sensitivity analysis excluding observations with missing workload ratings and applying complete-case analysis to the remaining model variables (Supplementary Table S3). For each unit shift, we calculated the number of RNs and patients and derived patient-to-RN ratios. Patient comorbidity was measured using the weighted Elixhauser summary score (Elixhauser et al., 1998; Menendez et al., 2014; Thompson et al., 2015; van Walraven et al., 2009). Unit-level comorbidity scores were computed as the mean Elixhauser score across all patients present during a shift. Patient turnover was quantified as admissions/transfers in (inbound) and discharges/transfers out (outbound) per shift (morning: 06:00–13:55; evening: 14:00–21:55; night: 22:00–05:55). Shift durations in Switzerland are typically 8–9 h, though deviations occur. Actual worked hours were categorized as: (1) undertime (<8 h); (2) typical (8–9 h); (3) moderate overtime (9–10 h); and (4) extreme overtime (>10 h).

### Statistical analysis

For aim 1, descriptive statistics were calculated by unit and shift. High workload was reported as n/N (%) of nurse-shift observations; mean and SD values were reported for patient-to-RN ratios, turnover, and comorbidity.

For aim 2, we examined criterion-related validity evidence of the single-item workload measure by testing its association with actual worktime. We used a multilevel logistic regression with high perceived workload as the binary outcome and worktime category as the main predictor (reference: 8–9 h).

For aim 3, we used a multilevel logistic regression to identify factors associated with high nurse-perceived workload. To account for systematic staffing patterns, an observed-to-expected (O/E) RN staffing ratio was calculated. Expected RN staffing was estimated separately for each unit using Poisson regression with shift type and weekday/weekend as predictors. Observed RN staffing was divided by the corresponding expected value. An O/E ratio of 1 indicates staffing at the expected level, while values below or above 1 indicate staffing below or above the expected level, respectively (Musy et al., 2021). Shift type and weekday/weekend were therefore incorporated through the expected staffing estimate rather than entered separately as fixed effects in the final workload model. The final model included the O/E staffing ratio, number of patients, mean Elixhauser score, inbound turnover, and outbound turnover as fixed effects, with random intercepts for nurses and units. Model performance was assessed using the area under the receiver operating characteristic curve (ROC-AUC) and the Brier score.

All models accounted for repeated observations per nurse nested within units. Random intercepts for units and nurses were included. Intraclass correlation coefficients (ICCs) were estimated for nurses (0.31, 95% CI 0.23–0.35) and units (0.01, 95% CI 0.00–0.03).

Finally, we explored whether high workload could be predicted from routine data using gradient boosting. The model included the O/E RN staffing ratio, number of patients, mean Elixhauser score, inbound turnover, and outbound turnover as predictors. The dataset was randomly split into training (80%) and test (20%) sets. The number of boosting rounds was selected using 5-fold cross-validation with early stopping. Discrimination was evaluated with ROC-AUC (DeLong), calibration with the Brier score, and predictor importance was examined.

All analyses were conducted in R, version 4.2.1 for MacOS (R Core Team, 2025). Predictive modelling used tidymodels and xgboost, ROC-AUC estimation via pROC, comorbidity scores with the comorbidity package, and ICCs with rptR (T. Chen et al., 2024; Gasparini, 2018; Kuhn and Wickham, 2020; Robin et al., 2011; Stoffel et al., 2017; Wickham et al., 2019).

### Ethical considerations

The study was outside the purview of the cantonal ethic committee based on the Swiss legislation on research with humans.

## Results

For aim 1, we described nurses’ workload perceptions and unit characteristics. After applying inclusion criteria, 131 RNs were included (Fig. 1). The dataset included 42,035 nurse-shift observations corresponding to 21,538 unit shifts. Workload ratings were missing for 10,687 (25.4%) nurse-shift observations; missingness varied across units from 16.5% to 47.7% and across shift types from 23.2% in morning shifts to 26.2% in evening shifts and 31.9% in night shifts (Supplementary Table S1). Because multiple nurses could rate the same unit shift, the number of nurse-shift ratings exceeded the number of unit shifts. In the primary analysis, 9,646 of 42,035 nurse-shift observations (22.9%) were classified as high workload. The proportion classified as high workload decreased from morning to evening to night and varied across units, ranging from 18.9% on unit 5 to 30.7% on unit 3 (Table 1, Supplementary Table S1).

**Fig. 1:**
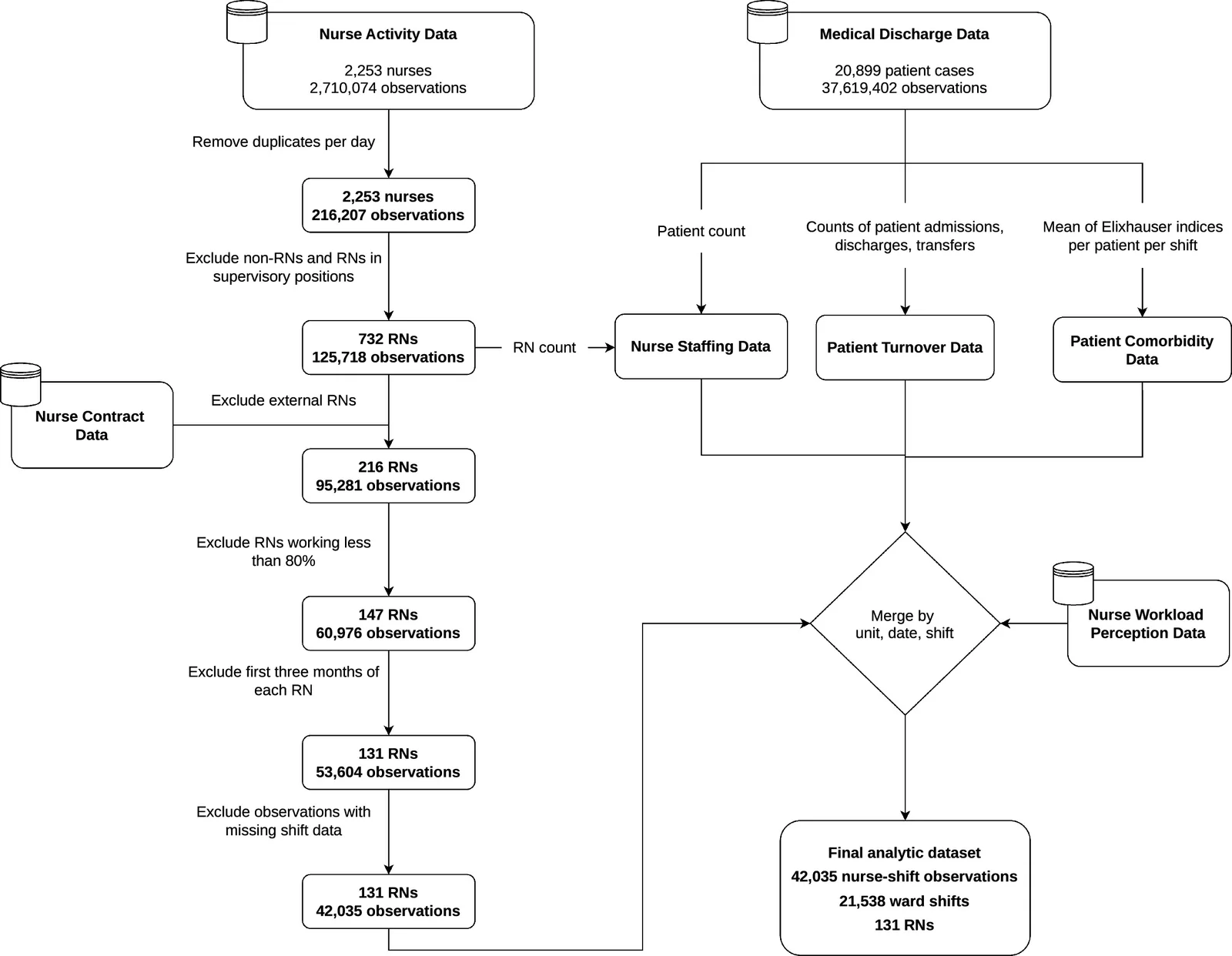
Flow chart of the data sources, exclusion steps, and data linkage process. The figure shows the derivation of the analytic sample from the nurse staffing system and discharge records, including the application of nurse eligibility criteria and linkage of shift-level staffing and patient data, resulting in 131 RNs, 42,035 nurse-shift observations, and 21,538 unit shifts.

**Table 1:** Descriptive overview of main variables by unit and shift type.

Unit	Shift	High workload	Patient to RN-ratio	Elixhauser (shift avg.)	Inbound turnover	Outbound turnover
		n/N (%)	Mean (SD)	Mean (SD)	Mean (SD)	Mean (SD)
1	morning	886/4188 (21.2%)	2.7 (±1.0)	12.4 (±3.1)	0.7 (±0.8)	1.4 (±1.3)
	evening	471/2197 (21.4%)	4.5 (±2.3)	12.7 (±3.1)	1.3 (±1.0)	1.3 (±1.3)
	night	122/1198 (10.2%)	10.6 (±4.2)	12.4 (±3.1)	0.8 (±0.8)	0.1 (±0.3)
2	morning	1244/3962 (31.4%)	2.5 (±0.9)	13.0 (±2.9)	0.7 (±0.8)	1.6 (±1.5)
	evening	510/2186 (23.3%)	3.8 (±1.5)	13.2 (±2.9)	1.5 (±1.2)	1.3 (±1.3)
	night	106/822 (12.9%)	11.9 (±4.7)	13.0 (±2.9)	0.7 (±0.8)	0.1 (±0.4)
3	morning	1227/4069 (30.2%)	2.6 (±1.0)	12.6 (±3.4)	0.6 (±0.7)	1.3 (±1.2)
	evening	677/2182 (31.0%)	4.0 (±2.0)	12.7 (±3.4)	1.1 (±1.0)	0.9 (±1.0)
	night	337/1050 (32.1%)	8.7 (±3.4)	12.8 (±3.4)	0.6 (±0.7)	0.1 (±0.3)
4	morning	1174/4995 (23.5%)	2.6 (±1.0)	13.1 (±3.1)	0.6 (±0.7)	1.4 (±1.3)
	evening	506/2533 (20.0%)	4.3 (±2.2)	13.3 (±3.2)	1.1 (±1.0)	0.9 (±1.0)
	night	230/1261 (18.2%)	9.3 (±3.8)	13.1 (±3.1)	0.6 (±0.7)	0.1 (±0.3)
5	morning	1153/5502 (21.0%)	3.1 (±1.0)	11.3 (±2.5)	0.8 (±0.8)	2.0 (±1.6)
	evening	682/3768 (18.1%)	3.8 (±1.3)	11.4 (±2.6)	1.7 (±1.2)	1.5 (±1.4)
	night	321/2122 (15.1%)	8.7 (±3.8)	11.3 (±2.4)	1.0 (±1.0)	0.1 (±0.3)

Patient-to-RN ratios increased from morning (mean 2.7, SD: 1.0) to evening (mean 4.0, SD: 1.9) to night shift (mean 9.6, SD: 4.1). While morning and evening ratios were similar across units at about three to four patients per RN, night shifts showed greater variation, from around nine patients per RN on units 3, 4 and 5 to twelve on unit 2 (Table 1). The overall mean Elixhauser score was 12.4 (SD: 3.1), corresponding to <10% in-hospital mortality risk(van Walraven et al., 2009). Differences across units were small, with the highest score on unit 4 and the lowest on unit 5, and variation across shifts was marginal. Inbound and outbound patient turnover showed clearer patterns, with admissions and transfers in most frequent in evening shifts (mean 1.4, SD: 1.1) and discharges and transfers out most common in mornings (mean 1.6, SD: 1.4). Most nurse-shifts (81%) lasted 8–9 h, 13% lasted 9–10 h, 0.5% exceeded 10 h, and 5% were shorter than 8 h. Overtime was most frequent on units 2 and 5 (20%) and least on unit 3 (7%).

For aim 2, we examined criterion-related validity evidence for the single-item workload measure by analysing its association with overtime. Results showed a clear pattern: shortened shifts were associated with substantially lower odds of high workload (OR 0.22, 95% CI: 0.18–0.26), whereas shifts longer than ten hours were associated with more than eight-fold higher odds of high workload (OR 8.22, 95% CI: 5.95–11.35) (Supplementary Table S2).

For aim 3, we identified factors associated with high workload using multilevel regression analysis. The strongest positive association was observed for inbound patient turnover during a shift (OR 1.29, 95% CI: 1.26–1.33), followed by the number of patients present (OR 1.24, 95% CI: 1.23–1.26). Higher RN staffing relative to expected staffing was associated with lower odds of high workload (OR 0.84, 95% CI: 0.78–0.91 per one-unit increase in the O/E ratio). A one-unit increase corresponds, for example, to moving from expected staffing (O/E = 1.0) to twice expected staffing (O/E = 2.0). A 0.1-point increase in the O/E ratio - for example, from 1.0 to 1.1, corresponding to staffing 10% above the expected level - was associated with approximately 2% lower odds of high workload (OR 0.98, 95% CI: 0.98–0.99). Comorbidity was weakly associated (OR 1.07, 95% CI: 1.06–1.08), and outbound patient turnover reached significance at the 0.05 level (Table 2). The regression model achieved an AUC of 0.80 and a Brier score of 0.14. A sensitivity analysis excluding observations with missing workload ratings and applying complete-case analysis to the remaining model variables included 31,338 nurse-shift observations and produced nearly identical estimates (Supplementary Table S3). An exploratory gradient boosting model showed limited discrimination in the held-out test set (AUC 0.59, 95% CI: 0.58–0.60; n = 8407) and a Brier score of 0.17. The O/E RN staffing ratio had the highest feature importance, followed by mean Elixhauser score and patient count; inbound and outbound turnover contributed less to the model (Fig. 2).

**Table 2:** Factors associated with nurse-perceived high workload in a logistic generalized linear mixed model and predictive performance.

	Odds Ratio [95% CI]
Intercept	0.002*** [0.001, 0.004]
O/E RN staffing ratio	0.841*** [0.779, 0.907]
Number of patients	1.243*** [1.227, 1.260]
Elixhauser (shift avg.)	1.066*** [1.057, 1.075]
Inbound turnover	1.290*** [1.255, 1.327]
Outbound turnover	1.023* [1.004, 1.043]
+ p < 0.1, * p < 0.05, ** p < 0.01, *** p < 0.001	
Number of observations	42,019
R2 Marg.	0.085
R2 Cond.	0.559
AIC	36,421.2
BIC	36,490.4
ICC	0.5
Area under the curve	0.80 [0.80–0.81]
Brier score	0.139

Notes: O/E = observed-over-expected; an O/E ratio of 1 indicates observed staffing equal to expected staffing. The OR is reported per one-unit increase in the O/E ratio.

Sixteen of 42,035 nurse-shift observations (0.04%) were excluded because of missing mean Elixhauser scores, resulting in an analytic sample of 42,019 observations.

**Fig. 2:**
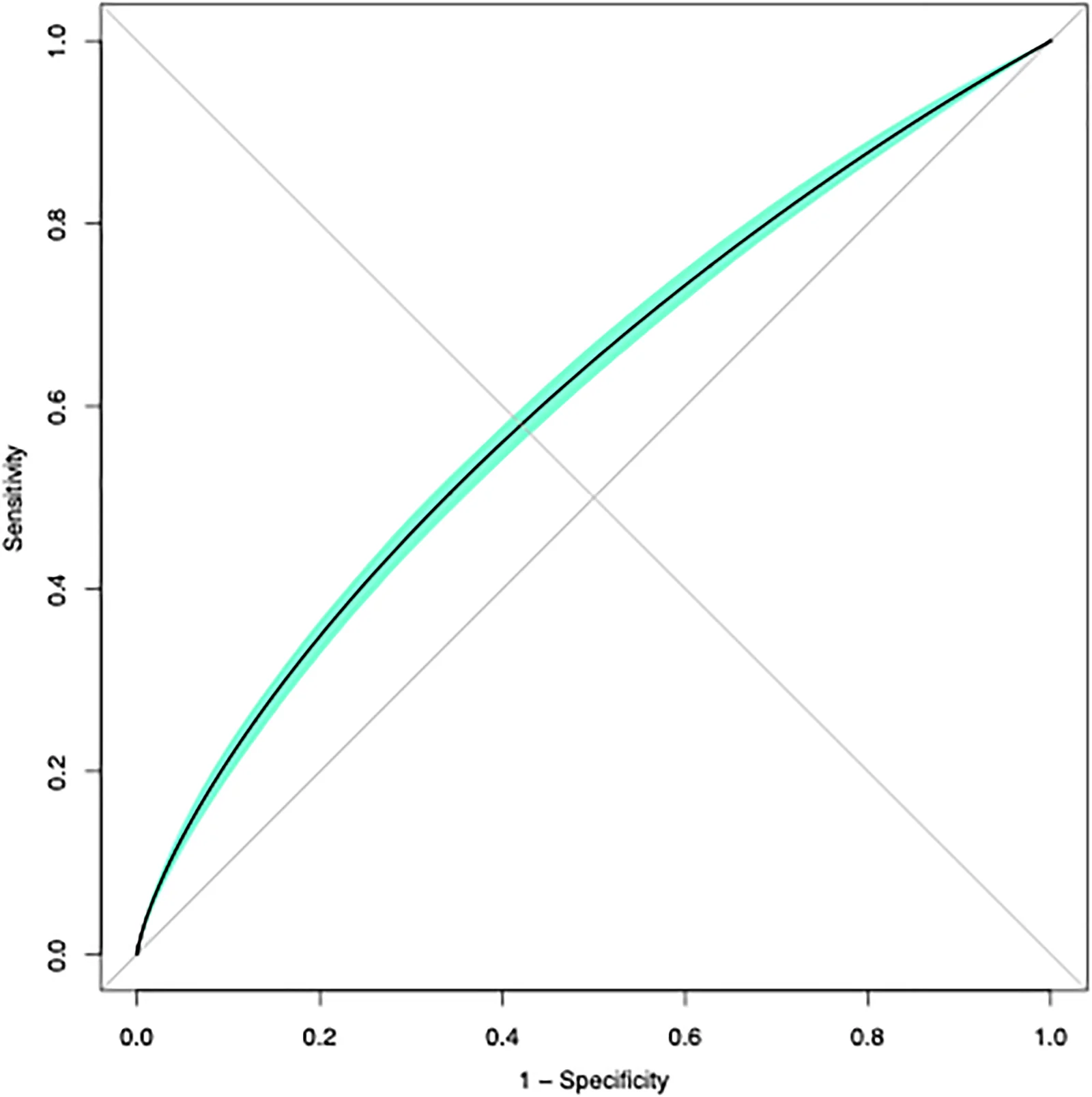
Receiver operating characteristic curve of the gradient-boosting model in the held-out test set (n = 8407). The area under the curve was 0.59 (95% CI: 0.58–0.60).

## Discussion

This study analysed nurse-perceived workload across five internal medicine units in a Swiss tertiary hospital using five years of shift-level data. In the primary analysis, 22.9% of nurse-shift observations were classified as high workload, with variation between units and across shift types. Morning shifts showed the highest proportion of high workload ratings. Higher RN staffing relative to expected staffing was associated with lower odds of high workload, while patient turnover and comorbidity were associated with higher odds. Workload perception clustered more strongly at the nurse than at the unit level. An exploratory gradient boosting model showed limited discrimination. The association between overtime and high perceived workload provides supportive criterion-related validity evidence for the single-item workload measure.

Nearly one-quarter of nurse-shift observations were classified as high workload, despite Switzerland’s comparatively favourable staffing levels and high healthcare expenditure. As these data predate the COVID-19 pandemic, they indicate that workload pressure is a structural feature of inpatient care. With workload recognised as a precursor to burnout, turnover, and reduced care quality (Galanis et al., 2025), these findings highlight a persistent challenge even in well-resourced settings. The higher proportion of high workload ratings during morning shifts likely reflects the clustering of direct care, admissions, and administrative activities during this period.

Higher RN staffing was moderately associated with lower perceived workload. The association between higher RN staffing relative to expected staffing and lower workload perception aligns with previous studies (Dhaini et al., 2022). The O/E ratio accounted for systematic differences in staffing by unit, shift type, and weekday/weekend and therefore captured deviations from expected staffing rather than absolute RN numbers. By adjusting observed staffing for structural patterns with an O/E estimator, our analysis provided a more nuanced assessment than simple patient-to-nurse ratios. Still, the magnitude of the association was limited, suggesting that ratios capture only part of the workload carried by nurses. Prior research shows that workload is shaped by non-linear dynamics, such as acuity, turnover, and skill mix (Leary and Punshon, 2019), and by relational and contextual factors (Oppel and Mohr, 2021).

Inbound patient turnover showed a particularly strong association with perceived workload, with admissions and inbound transfers associated with substantially higher odds of high workload. This finding is consistent with studies linking admissions to increased task load, workflow disruption, and documentation burden (Ivziku et al., 2022; VanFosson et al., 2017). Outbound turnover was only weakly associated, possibly reflecting lower care intensity prior to discharge. Patient comorbidity showed a modest association, which may reflect the limitations of the Elixhauser score in capturing real-time care intensity in internal medicine. More granular measures, such as functional status or acuity assessments, may better reflect care needs (Mueller et al., 2010).

Variation in workload perception was explained more by differences between individual nurses than by differences between units. This may reflect personal factors such as workload tolerance or clinical experience, but the small number of units limits conclusions. Similar patterns have been reported in other studies with few units (Dhaini et al., 2022), while other research highlights the influence of unit-level factors such as leadership and teamwork (MacPhee et al., 2017; van den Oetelaar et al., 2021; Yanchus et al., 2017). Both individual and contextual factors are therefore relevant when interpreting workload.

The gradient boosting model showed little predictive performance, indicating that nurse-perceived workload was difficult to predict from the available routine data. Emotional strain, cumulative demands, and unpredictability may be unlikely to be captured in such datasets. While some studies have achieved higher predictive accuracy using patient classification systems (Meredith et al., 2025), these approaches do not fully capture the broader influences that shape perceived workload.

The observed association between overtime and perceived workload provides supportive criterion-related validity evidence for the single-item workload measure. More comprehensive tools such as the NASA Task Load Index (NASA-TLX) (Hart, 2006) or the Questionnaire on the Experience and Evaluation of Work 2.0 (QEEW 2.0) (Veldhoven et al., 2015) provide detailed multidimensional assessments but are resource-intensive and impractical for continuous use (Hoogendoorn et al., 2020; Racy et al., 2021). In contrast, single-item ratings embedded in surveys or information systems impose minimal burden and can be readily integrated into routine monitoring (Decock et al., 2020; Diamantopoulos et al., 2012; Laerkner et al., 2016).

While scepticism toward nurses’ subjective workload ratings may be understandable, a growing body of evidence supports their validity. Such ratings have been associated with lower inpatient mortality (Smeds-Alenius et al., 2016), nurse outcomes including shift satisfaction (Abed Al Ahad et al., 2022), and care rationing (Dhaini et al., 2020; Hegney et al., 2019), a known predictor of patient outcomes (Kalánková et al., 2020; Schubert et al., 2008). Other research has shown that standard metrics may underestimate workload early in patient care and that cumulative workload over time contributes to sustained perceptions of high workload (Chan et al., 2023, Chen et al., 2024). Together, this evidence underlines the importance of integrating both subjective and objective measures when assessing workload.

Overall, this study highlights the limitations of relying solely on patient-to-nurse ratios or other objective indicators, which often fail to capture patient complexity, turnover, or non-patient-related activities (Griffiths et al., 2018; Musy et al., 2020; Twigg and Duffield, 2009). Prior studies have shown that it complements ratio-based measures and may provide a more accurate representation of workload (Dhaini et al., 2022; Fischbacher et al., 2024). Perceived workload reflects additional aspects of demand and variability between nurses. Considering both objective and subjective measures provides a more complete picture and may support more responsive workforce planning.

## Limitations

This study has several strengths, including the use of five years of routine shift-level data, integration of objective and subjective workload measures, and the application of multilevel modelling. Limitations include the absence of individual-level nurse characteristics, which prevented stratified analyses; and a substantial proportion of missing workload ratings. Coding missing ratings as not high workload in the primary analysis assumes that missing ratings did not indicate high workload, although sensitivity analysis excluding these observations produced nearly identical estimates. The single-item workload measure has not undergone formal psychometric validation, and overtime is an imperfect external indicator of workload, as longer worktime may also reflect organisational or scheduling factors and high workload does not necessarily result in overtime. Furthermore, the analysis was restricted to five units in one hospital, limiting generalisability.

## Conclusions

In conclusion, nurse-perceived workload was reported in nearly one-quarter of nurse-shift observations and was most strongly associated with patient turnover and staffing. Even in a well-resourced healthcare system, workload pressures persist, suggesting that current staffing models may not fully address the demands nurses face. Subjective workload ratings represent a pragmatic, low-burden indicator that complements objective measures and should be considered in workforce planning to support sustainable, high-quality care.

## Declaration of generative AI and AI-assisted technologies in the manuscript preparation process

During the preparation of this work the author(s) used ChatGPT (GPT-5, OpenAI) in order to improve the clarity, grammar, and overall language quality of the text. After using this tool/service, the author(s) reviewed and edited the content as needed and take(s) full responsibility for the content of the published article.

## Funding

This research did not receive any specific grant from funding agencies in the public, commercial, or not-for-profit sectors.

## CRediT authorship contribution statement

**Michael Ketzer:** Writing – original draft, Formal analysis, Data curation, Conceptualization. **Sarah N. Musy:** Writing – review & editing, Project administration, Data curation, Conceptualization. **Christos T. Nakas:** Writing – review & editing, Methodology, Formal analysis. **Michael Simon:** Writing – review & editing, Supervision, Methodology.

## Declaration of competing interest

The authors declare that they have no known competing financial interests or personal relationships that could have appeared to influence the work reported in this paper.
