# Supplementary material for: Nurse-perceived workload, staffing, and patient turnover: An analysis of longitudinal routine data
Source: Int J Nurs Stud Adv. 2026 Sep 5;11:100671. doi: 10.1016/j.ijnsa.2026.100671 (PMC13663898; doi:10.1016/j.ijnsa.2026.100671)
Supplement: Supplementary file 1 [file mmc1.docx]

**Supplement: Additional Descriptive Information and Regression Results**

|  | SAB (workload rating) | | | | | |
| --- | --- | --- | --- | --- | --- | --- |
|  | -2  (very low workload) | -1  (low workload) | 0  (neutral workload) | 1  (high workload) | 2  (very high workload) | NA  (Missing rating) |
| Unit |  |  |  |  |  |  |
| 1 | 37 (0.5%) | 407 (5.4%) | 4193 (55.3%) | 1192 (15.7%) | 287 (3.8%) | 1467 (19.3%) |
| 2 | 12 (0.2%) | 117 (1.7%) | 3276 (47.0%) | 1506 (21.6%) | 354 (5.1%) | 1705 (24.5%) |
| 3 | 1 (0.0%) | 54 (0.7%) | 3798 (52.0%) | 1832 (25.1%) | 409 (5.6%) | 1207 (16.5%) |
| 4 | 8 (0.1%) | 170 (1.9%) | 2510 (28.6%) | 1262 (14.4%) | 648 (7.4%) | 4191 (47.7%) |
| 5 | 25 (0.2%) | 413 (3.6%) | 6681 (58.6%) | 1842 (16.2%) | 314 (2.8%) | 2117 (18.6%) |
| Shift type |  |  |  |  |  |  |
| Morning | 41 (0.2%) | 561 (2.5%) | 11166 (49.2%) | 4525 (19.9%) | 1159 (5.1%) | 5264 (23.2%) |
| Evening | 23 (0.2%) | 412 (3.2%) | 6218 (48.3%) | 2208 (17.2%) | 638 (5.0%) | 3367 (26.2%) |
| Night | 19 (0.3%) | 188 (2.9%) | 3074 (47.6%) | 901 (14.0%) | 215 (3.3%) | 2056 (31.9%) |
| Total | 83 (0.2%) | 1161 (2.8%) | 20458 (48.7%) | 7634 (18.2%) | 2012 (4.8%) | 10687 (25.4%) |

Supplementary Table S1: Distribution of nurse-perceived workload ratings, including missing ratings, by unit and shift type

|  | **Odds Ratio [95% CI]** |
| --- | --- |
| (Intercept) | **0.116***** [0.086, 0.156] |
| Undertime | **0.219***** [0.183, 0.262] |
| Moderate overtime | **2.189***** [2.033, 2.356] |
| Extreme overtime | **8.221***** [5.954, 11.352] |
| + p < 0.1, * p < 0.05, ** p < 0.01, *** p < 0.001 | |
| Num.Obs. | 42035 |
| R2 Marg. | 0.033 |
| R2 Cond. | 0.496 |
| AIC | 36852.5 |
| BIC | 36904.3 |
| ICC | 0.5 |

Supplementary Table S2: Association between nurse-perceived high workload and reported work hours (reference: typical shift duration)

|  | **Odds Ratio [95% CI]** |
| --- | --- |
| Intercept | **0.006***** [0.003, 0.014] |
| O/E estimator RN Staffing | **0.819***** [0.757, 0.887] |
| Number of patients | **1.256***** [1.239, 1.273] |
| Elixhauser (shift avg.) | **1.071***** [1.061, 1.080] |
| Inbound turnover | **1.305***** [1.268, 1.344] |
| Outbound turnover | **1.023*** [1.003, 1.043] |
| + p < 0.1, * p < 0.05, ** p < 0.01, *** p < 0.001 | |
| Num.Obs. | 31338 |
| R2 Marg. | 0.125 |
| R2 Cond. | 0.421 |
| AIC | 33127.1 |
| BIC | 33193.9 |
| ICC | 0.3 |
| Note: Of 42,035 nurse-shift observations, 31,348 had a non-missing workload rating; 10 additional observations were excluded because of missing data in other model variables, resulting in an analytic sample of 31,338. | |

Supplementary Table S3: Sensitivity analysis excluding missing workload ratings: factors associated with nurse-perceived high workload in a logistic generalized linear mixed model
